# Gastric Sarcoidosis: A Rare Clinical Presentation

**DOI:** 10.1155/2013/260704

**Published:** 2013-12-04

**Authors:** Hemasri Tokala, Karthik Polsani, Jagadeesh K. Kalavakunta

**Affiliations:** ^1^Department of Internal Medicine, Michigan State University, 804 Service Road, East Lansing, MI 48824, USA; ^2^Indiana University Health Physicians, Community Howard Regional Health, Kokomo, IN, USA

## Abstract

Gastrointestinal (GI) sarcoidosis is a very rare disease, which clinically presents along with systemic disease or as an isolated finding. Gastric sarcoidosis is the most common form of GI sarcoidosis. Symptomatic gastric sarcoidosis is rare and only few case reports have been described in the literature with well-documented histological evidence of noncaseating granulomas. We present an interesting case of gastric sarcoidosis in a 39-year-old Caucasian man with symptoms of epigastric pain and profound weight loss. His endoscopic gastric mucosal biopsies revealed noncaseating granulomas consistent with gastric sarcoidosis. Treatment with oral steroids alleviated his symptoms with no recurrence in 2 years. Gastric sarcoidosis should be considered in patients with history of sarcoidosis and GI symptoms.

## 1. Introduction

Sarcoidosis is a chronic noncaseating granulomatous systemic inflammatory disease. Even though it was first described by Sir Jonathan Hutchinson 140 years ago, to date the etiology is unclear [1]. It can involve any organ, with pulmonary involvement being the most common. Gastrointestinal (GI) involvement is very rare and may present along with systemic disease or as an isolated finding. Gastric sarcoidosis, first described by Schaumann in 1936, is the most common form of GI tract sarcoidosis [2]. Symptomatic GI involvement occurs only in 0.1 to 0.9% of patients with systemic disease [3]. In the literature there are only 26 reported cases of symptomatic gastric sarcoidosis with well-documented histological evidence of noncaseating granulomas consistent with sarcoidosis [4].

## 2. Case Presentation 

**Figure 1:**
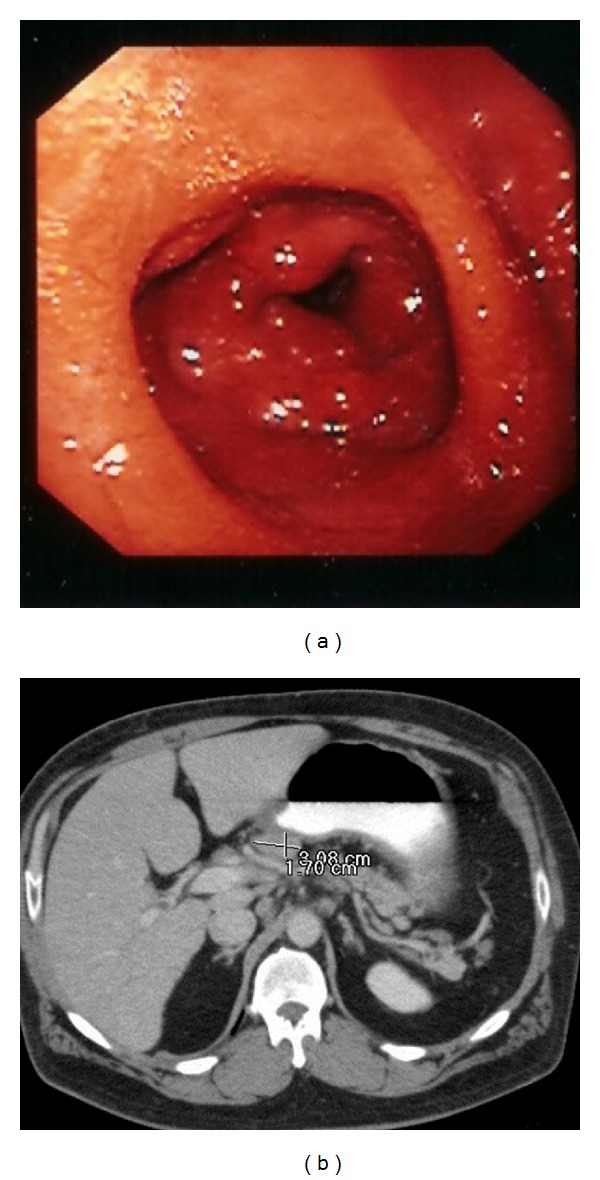
(a) Upper gastrointestinal endoscopy showing linitis plastic-like appearance and diffuse erythema. (b) Computer tomography showing the intra-abdominal adenopathy.

**Figure 2:**
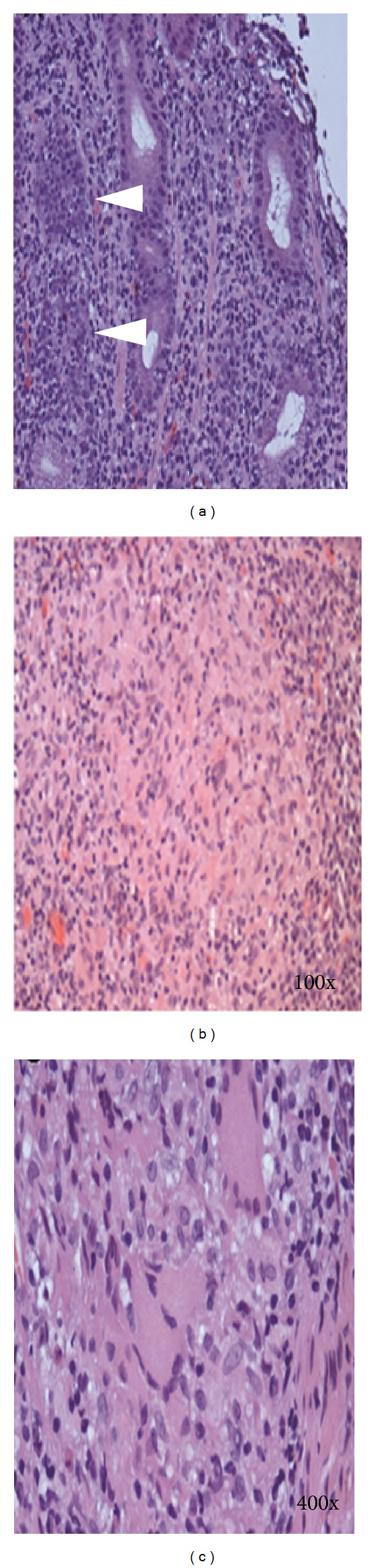
Histopathology of the upper gastrointestinal endoscopic biopsies revealing several small noncaseating epitheloid cell granulomas (arrow heads) and without any evidence of dysplasia or intestinal metaplasia (b) (100x) (c) (400x) showing the noncaseating granuloma.

A 39-year-old Caucasian man who is a race car driver by profession presented to the emergency department with a six-month history of nausea, vomiting, and profound weight loss along with one-month history of progressively increasing epigastric pain. His past history included incidental diagnosis of sarcoidosis in spleen and gall bladder one year ago when he met with a motor vehicle accident and underwent elective cholecystectomy and splenectomy. Physical examination was remarkable for mild tenderness in the epigastric region. Rest of the examination was unremarkable. Complete blood counts, comprehensive metabolic panel, and chest X-ray were normal. Computed tomography scan of the abdomen and pelvis revealed extensive adenopathy in the mesentery and retroperitoneum (Figure 1(b)). Esophagogastroduodenoscopy done during the hospital stay was significant for poor gastric insufflation and the wall of the stomach looked very rigid with diffuse erythema resembling linitis plastica, without any evidence of ulcers or tumors (Figure 1(a)). Random biopsies were taken from different sections of stomach. Histopathology revealed chronic and acute inflammation with several small noncaseating granulomas composed of epithelioid, histiocytes, and multinucleated giant cells without any evidence of dysplasia or intestinal metaplasia (Figure 2). Biopsies were stained for *Helicobacter pylori*, and *Mycobacterium* (AFB stain) and fungal organisms were all negative. Other laboratory workup to rule out the differential causes also came negative. With history of sarcoidosis, symptoms, and lab workup along with the histological findings the diagnosis was confirmed as gastric sarcoidosis. Prednisone 60 mg per day was started and he had alleviation of symptoms within four days. He was placed on a tapering dose of prednisone for a period of 6 months with no recurrence of symptoms in 2 years.

## 3. Discussion

Most gastric sarcoidosis cases are asymptomatic. It mainly affects the antrum of the stomach and symptoms can be related to the ulceration of the gastric mucosa or due to the diffuse infiltration and fibrosis of the mucosa leading to the narrowing of the gastric lumen. Epigastric pain (75%) is the most common symptom. Other symptoms are early satiety, nausea, vomiting, hematemesis, melena, and weight loss [5].

Gastrointestinal sarcoidosis can present either as an ulcer or as diffuse involvement resembling linitis plastica. Endoscopy along with biopsies is critical in the diagnosis of the gastric sarcoidosis. Depending on the pathology, endoscopic findings can differ. With diffuse infiltration of the mucosa it can appear as linitis plastica (leather bottle) as in our case. In other pathologies we can see mucosal ulcers with or without erythema and polypoid/nodular lesions (due to granulomas). In asymptomatic patients the gastric mucosa can be normal and can be easily overlooked.

Gastric sarcoidosis is unique as it can mimic other GI diseases in presentation and its diagnosis requires proper interpretation of the biopsies as many other etiologies can present with noncaseating granulomas. In the differential diagnosis we need to consider peptic ulcer disease, Ménétrier disease, hypertrophic gastritis, *Mycobacterium*, syphilis, histoplasmosis, gastric cancer, lymphoma, Langerhans cell histiocytosis, foreign body reaction, and Crohn's and Whipple's diseases.

Treatment depends on the symptoms. Asymptomatic patients do not need any specific therapy. Steroids are the initial treatment of choice in symptomatic patients with or without proton pump inhibitors depending on the presence of ulcers. In a recent report 66% of patients had alleviation of symptoms with steroids [6]. Role of immunosuppressive therapy is not well defined in the literature. Surgery might be useful when there is severe gastric lumen narrowing or obstruction. In our patient, steroids alleviated the symptoms within four days with no recurrence of symptoms in 2 years.

## 4. Conclusion

Gastric sarcoidosis should be considered in patients with history of sarcoidosis and complaints of epigastric pain and unexplained weight loss.
